# Occurrence of breast cancer subtypes in adolescent and young adult women

**DOI:** 10.1186/bcr3156

**Published:** 2012-03-27

**Authors:** Theresa HM Keegan, Mindy C DeRouen, David J Press, Allison W Kurian, Christina A Clarke

**Affiliations:** 1 Cancer Prevention Institute of California, 2201 Walnut Ave, Suite 300, Fremont, CA 94538, USA; 2 Division of Epidemiology, Department of Health Research and Policy, Stanford University School of Medicine, Stanford, CA 94305, USA; 3 Department of Medicine, Stanford University School of Medicine, 300 Pasteur Drive, Stanford, CA 94305, USA

## Abstract

**Introduction:**

Breast cancers are increasingly recognized as heterogeneous based on expression of receptors for estrogen (ER), progesterone (PR), and human epidermal growth factor receptor 2 (HER2). Triple-negative tumors (ER^-^/PR^-^/HER2^-^) have been reported to be more common among younger women, but occurrence of the spectrum of breast cancer subtypes in adolescent and young adult (AYA) women aged between 15 and 39 years is otherwise poorly understood.

**Methods:**

Data regarding all 5,605 AYA breast cancers diagnosed in California during the period 2005 to 2009, including ER and PR status (referred to jointly as hormone receptor (HR) status) and HER2 status, was obtained from the population-based California Cancer Registry. Incidence rates were calculated by subtype (triple-negative; HR^+^/HER2^-^; HR^+^/HER2^+^; HR^-^/HER2^+^), and logistic regression was used to evaluate differences in subtype characteristics by age group.

**Results:**

AYAs had higher proportions of HR^+^/HER2^+^, triple-negative and HR^-^/HER2^+ ^breast cancer subtypes and higher proportions of patients of non-White race/ethnicity than did older women. AYAs also were more likely to be diagnosed with stage III/IV disease and high-grade tumors than were older women. Rates of HR^+^/HER2^- ^and triple-negative subtypes in AYAs varied substantially by race/ethnicity.

**Conclusions:**

The distribution of breast cancer subtypes among AYAs varies from that observed in older women, and varies further by race/ethnicity. Observed subtype distributions may explain the poorer breast cancer survival previously observed among AYAs.

## Introduction

Breast cancer is the most frequently diagnosed cancer among adolescent and young adult (AYA) women 15 to 39 years of age [1]. Currently, AYA breast cancer accounts for approximately 14% of all AYA cancer diagnoses and 7% of all breast cancer diagnoses [1,2]. Evidence suggests that AYA breast cancer may be etiologically as well as clinically distinct from breast cancer in older women [2]. When breast cancer occurs in AYAs, it differs from that occurring in older women in several ways: a worse prognosis and more-aggressive phenotype, higher proportions of high-grade and later stage tumors, lower estrogen receptor (ER) positivity, and overexpression of human epidermal growth factor receptor 2 (HER2) [1,3,4]. Understanding differences between AYA cancers and those occurring in older populations has been set forth by the National Cancer Institute and LIVESTRONG Young Adult Alliance as important in light of stalled progress in improving outcomes among AYAs with cancer [5].

We recently reported in a large, population-based series from California that the hormone receptor negative, HER2 positive (HR^-^/HER2^+^) and triple-negative (ER negative/progesterone receptor (PR) negative/HER2 negative) tumors were more common in younger women [4,6]. Other California-based analyses have found a higher incidence rate of triple-negative breast cancer in young (younger than 44 years) Blacks compared with Whites or Hispanics (2004 to 2006) [7] and that young age was associated with triple-negative breast cancer in 1999 to 2003 [8] and 1999 to 2004 [9] data. The Carolina Breast Cancer Study also found triple-negative breast cancer to be more common in premenopausal than postmenopausal women [10]. However, besides triple-negative breast cancer [7], no previous studies have reported incidence rates by molecular breast cancer subtypes in AYAs. Therefore, the better to understand the occurrence of AYA breast cancer subtypes, we took advantage of HER2 data recently available for breast cancers occurring in the large and diverse California population from 2005 to 2009. In addition, we compared the subtype distribution and demographic and tumor characteristics in AYAs with those in older premenopausal (40 to 49 years) and postmenopausal (50+ years) women.

## Methods

### Cancer cases

We obtained from the California Cancer Registry (CCR) information about all female California residents diagnosed with an invasive breast cancer (International Classification of Disease for Oncology, 3^rd ^Edition, (ICD-O-3) site codes C50.0-50.9) during the period January 1, 2005, through December 31, 2009. Individual informed consent was not obtained, as the analysis was based on state-mandated cancer registry data. For each breast cancer case, we obtained information routinely abstracted from the medical record on age at diagnosis, race/ethnicity (Hispanic, non-Hispanic White, non-Hispanic Black, and non-Hispanic Asian/Pacific Islander, hereafter referred to as "White", "Black", "Hispanic", and "Asian"), AJCC stage at diagnosis (I, II, III, IV, or unstaged/not applicable (NA)), tumor size (≤2 cm, > 2 cm, microinvasion, diffuse, or unknown), grade (low, high, or unknown), ER, PR, and HER2 tumor-expression status, sequence number (first primary or non-first primary), and prior cancer (no, yes, or unknown). The CCR has collected information on ER and PR since 1990 and on HER2 since 1999 [8]. Before the year 2005, 41% of cases lacked HER2 data, but data completeness has increased to at least 83% since that time. Because of the initial reduced reliability of HER2-receptor status [11] and data completeness, we limited our analyses to between 2005 and 2009. ER and PR were evaluated with dextran-coated charcoal assays or immunohistochemistry (IHC); HER2 was tested with IHC or fluorescence *in situ *hybridization. Each marker was reported as positive, negative, borderline, not tested, not recorded, or unknown, based on the test performed by the laboratory at the reporting facility [12].

Of the 141,002 female breast cancer cases 15 years or older diagnosed between 2005 and 2009 in California, we excluded cases with *in situ *breast cancer (*n *= 27,276), inflammatory carcinoma (*n *= 697), Paget disease (*n *= 26), mammographic or xerographic diagnosis only (*n *= 383), and death certificate only (*n *= 363). The resulting study population (*N *= 112,256) included 5,605 women aged 15 to 39 years, 19,776 women aged 40 to 49 years, and 86,875 women older than 50 years at diagnosis.

### Categorization of breast cancer subtypes

Breast cancer subtypes were categorized according to tumor expression of ER, PR, and HER2. HR^+^/HER2^- ^was defined as ER or PR positive and HER2 negative; HR^+^/HER2^+ ^as ER or PR positive and HER2 positive; HR^-^/HER2^+ ^as ER and PR negative and HER2 positive; and triple-negative as ER, PR, and HER2 negative [11,13,14].

### Population denominator data

We obtained population counts by sex, race/ethnicity, and 5-year age group for the state of California from the 2000 Census Summary File 3 (SF-3) [15]. Data from the 20% Integrated Public-Use Microdata Sample of the Census also were used to estimate age-specific population counts for Hispanics [16] by smoothing with a spline-based function.

### Statistical analysis

SEER*Stat software [17] was used to compute age-adjusted incidence rates (standardized to the 2000 US standard million population) and 95% confidence intervals (CIs) for invasive breast cancer. We calculated incidence-rate ratios (IRRs) comparing rates by race/ethnicity, with Whites as the reference. To evaluate differences in the clinical characteristics of breast cancer subtypes by age group (15 to 39 years versus 40 to 49 years; 15 to 39 years versus older than 50 years), and we used logistic regression to calculate odds ratios (OR) and associated 95% confidence intervals (CIs). Models included race/ethnicity, year of diagnosis, stage at diagnosis, grade, and first primary cancer, and were analyzed by using SAS version 9.2 (Cary, NC, USA). This project was approved by the institutional review board of the Cancer Prevention Institute of California.

## Results

**Figure 1:**
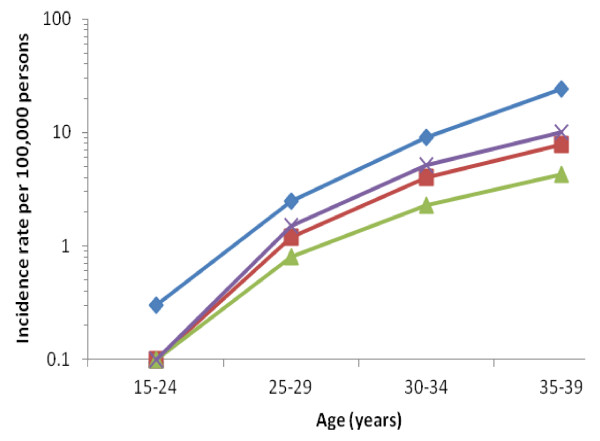
**Age-specific incidence rates of breast cancer by subtypes among California women aged 15 to 39 years, 2005-2009**. Hormone receptor (HR)-positive and human epidermal growth factor receptor 2 (HER2)-negative (diamond), HR^+^/HER2^+ ^(square), HR^-^/HER2^+ ^(triangle), and triple-negative (X).

The overall, age-adjusted incidence rate for breast cancer among all AYAs was 18.9 per 100,000 women (95% CI, 18.4 to 19.4). Incidence of breast cancer for all subtypes increased rapidly between 15 and 39 years of age (Figure 1). Table 1 shows characteristics of AYA breast tumors diagnosed. 62.8% of AYAs were diagnosed between 35 to 39 years of age (Table 1). HR^+^/HER2^- ^was the most commonly diagnosed AYA subtype (41.1%), followed by triple-negative (19.1%), HR^+^/HER2^+ ^(15.0%) and HR^-^/HER2^+ ^(8.7%). Most AYA breast cancer patients were of White (42.7%) or Hispanic (32.1%) race/ethnicity. The highest proportion of stage III/IV disease occurred for the HR^-^/HER2^+ ^subtype. A higher proportion of AYAs with triple-negative and HR^-^/HER2^+ ^subtypes presented with high-grade disease than did those diagnosed with the HR^+^/HER2^- ^subtype. Nearly 7% of AYA breast cancer patients had a prior cancer (any type) before this breast cancer.

**Table 1:** Demographic and clinical characteristics of adolescents and young adults with breast cancer by subtype,^a ^2005 to 2009

	Total	**HR^+^/HER2**^**-a**^		HR^+^/HER2^+a^		HR^-^/HER2^+a^		**Triple-negative**^a^		Unclassified	
	(*N *= 5,605)	(*n *= 2,306)		(*n *= 843)		(*n *= 485)		(*n *= 1,073)		(*n *= 898)	
Characteristics	*N*	*n*	Col%	*n*	Col%	*n*	Col%	*n*	Col%	*n*	Col%
Age group (years)											
15-24	97	36	(1.6)	11	(1.3)	12	(2.5)	11	(1.0)	27	(3.0)
25-29	482	161	(7.0)	81	(9.6)	53	(10.9)	98	(9.1)	89	(9.9)
30-34	1,507	557	(24.2)	246	(29.2)	144	(29.7)	319	(29.7)	241	(26.8)
35-39	3,519	1,552	(67.3)	505	(59.9)	276	(56.9)	645	(60.1)	541	(60.2)
Race/ethnicity											
White	2,394	1,050	(45.5)	347	(41.2)	198	(40.8)	430	(40.1)	369	(41.1)
Black	421	135	(5.9)	66	(7.8)	39	(8.0)	117	(10.9)	64	(7.1)
Hispanic	1,801	678	(29.4)	271	(32.2)	166	(34.2)	394	(36.7)	292	(32.5)
Asian	917	423	(18.3)	152	(18.0)	79	(16.3)	116	(10.8)	147	(16.4)
Unknown/other	72	20	(0.9)	7	(0.8)	^b^		16	(1.5)	26	(2.9)
Year of diagnosis											
2005	1,134	367	(15.9)	157	(18.6)	101	(20.8)	226	(21.1)	283	(31.5)
2006	1,133	444	(19.3)	192	(22.8)	106	(21.9)	212	(19.8)	179	(19.9)
2007	1,074	463	(20.1)	145	(17.2)	100	(20.6)	207	(19.3)	159	(17.7)
2008	1,205	540	(23.4)	168	(19.9)	103	(21.2)	239	(22.3)	155	(17.3)
2009	1,059	492	(21.3)	181	(21.5)	75	(15.5)	189	(17.6)	122	(13.6)
AJCC stage at diagnosis											
I	1,435	722	(31.3)	183	(21.7)	94	(19.4)	212	(19.8)	224	(24.9)
II	2,425	956	(41.5)	365	(43.3)	199	(41.0)	541	(50.4)	364	(40.5)
III	1,102	430	(18.6)	214	(25.4)	126	(26.0)	213	(19.9)	119	(13.3)
IV	327	127	(5.5)	47	(5.6)	45	(9.3)	63	(5.9)	45	(5.0)
Unstaged/NA	316	71	(3.1)	34	(4.0)	21	(4.3)	44	(4.1)	146	(16.3)
Grade											
Low	2,222	1,370	(59.4)	341	(40.5)	104	(21.4)	100	(9.3)	307	(34.2)
High	3,022	849	(36.8)	466	(55.3)	356	(73.4)	939	(87.5)	412	(45.9)
Unknown	361	87	(3.8)	36	(4.3)	25	(5.2)	34	(3.2)	179	(19.9)
Tumor size											
≤2 cm	2,165	1,056	(45.8)	326	(38.9)	157	(32.4)	320	(29.8)	306	(34.0)
> 2 cm	3,093	1,177	(51.0)	479	(57.1)	284	(58.6)	705	(65.7)	448	(49.8)
Microinvasion	56	15	(0.7)	7	(0.8)	8	(1.7)	^b^		26	(2.9)
Diffuse	38	10	(0.4)	^b^		12	(2.5)	9	(0.8)	^b^	
Unknown	253	48	(2.1)	27	(3.2)	24	(5.0)	39	(3.6)	115	(12.8)
Lymph node involvement											
No	2,761	1,155	(50.1)	358	(42.5)	189	(39.0)	566	(52.8)	493	(54.9)
Yes	2,707	1,123	(48.7)	472	(56.0)	286	(59.0)	491	(45.8)	335	(37.3)
Unknown	137	28	(1.2)	13	(1.5)	10	(2.1)	16	(1.5)	70	(7.8)
Prior cancer											
First primary	5,226	2,169	(94.1)	796	(94.4)	459	(94.6)	993	(92.5)	832	(92.7)
Non-first primary	379	137	(5.9)	47	(5.6)	26	(5.4)	80	(7.5)	66	(7.3)

^a^Human epidermal growth factor receptor 2 (HER2), hormone receptor (HR), triple-negative (estrogen-receptor negative, progesterone-receptor negative, HER2-). ^b^Statistic not displayed because of five or fewer cases.

Substantial racial/ethnic differences were found in AYA breast cancer. Compared with Whites, Blacks were 24% less likely to have the HR^+^/HER2^- ^subtype and 61% more likely to have the triple-negative subtype. Unlike in the other racial/ethnic groups, in which the incidence of HR^+^/HER2^- ^is higher than triple-negative breast cancer, Blacks had a similar incidence of HR^+^/HER2^- ^and triple-negative subtypes. In addition, Hispanics were 35% less likely to be diagnosed with the HR^+^/HER2^- ^subtype, and Asians were 36% less likely to be diagnosed with the triple-negative subtype than were Whites (Table 2).

**Table 2:** Age-adjusted breast cancer incidence rates among adolescent and young adults by subtype,^a ^California, 2005 to 2009

Characteristics	Total **rate (95% CI)**^b^	HR^+^/HER2^-^ rate (95% CI)	HR^+^/HER2^+^ rate (95% CI)	HR^-^/HER2^+^ rate (95% CI)	Triple-negative rate (95% CI)
Age group (years)					
15-24	0.7 (0.6-0.9)	0.3 (0.2-0.4)	0.1 (0.0-0.2)	0.1 (0.0-0.2)	0.1 (0.0-0.2)
25-29	7.4 (6.8-8.1)	2.5 (2.1-2.9)	1.2 (1.0-1.5)	0.8 (0.6-1.1)	1.5 (1.2-1.8)
30-34	24.4 (23.2-25.7)	9.0 (8.3-9.8)	4.0 (3.5-4.5)	2.3 (2.0-2.7)	5.2 (4.6-5.8)
35-39	54.5 (52.7-56.3)	24.0 (22.8-25.2)	7.8 (7.1-8.5)	4.3 (3.8-4.8)	10.0 (9.2-10.8)
Race/ethnicity					
White	21.0 (20.2-21.9)	9.2 (8.7-9.8)	3.0 (2.7-3.4)	1.7 (1.5-2.0)	3.8 (3.4-4.1)
Black	21.8 (19.7-23.9)	7.0 (5.9-8.3)	3.4 (2.6-4.3)	2.0 (1.4-2.8)	6.1 (5.0-7.3)
Hispanic	15.8 (15.1-16.6)	6.0 (5.6-6.5)	2.4 (2.1-2.7)	1.4 (1.2-1.7)	3.4 (3.1-3.8)
Asian	19.4 (18.1-20.7)	9.0 (8.2-9.9)	3.2 (2.7-3.8)	1.6 (1.3-2.1)	2.4 (2.0-2.9)
Incidence rate ratio					
White	Reference	Reference	Reference	Reference	Reference
Black	1.04 (0.93-1.16)	0.76 (0.63-0.91)	1.11 (0.85-1.44)	1.18 (0.83-1.66)	1.61 (1.31-1.97)
Hispanic	0.75 (0.70-0.81)	0.65 (0.54-0.79)	0.78 (0.60-1.02)	0.83 (0.58-1.17)	0.91 (0.74-1.12)
Asian	0.92 (0.84-1.01)	0.98 (0.87-1.10)	1.05 (0.86-1.28)	0.95 (0.73-1.25)	0.64 (0.52-0.79)
AJCC Stage at diagnosis					
I	4.9 (4.6-5.1)	2.5 (2.3-2.6)	0.6 (0.5-0.7)	0.3 (0.3-0.4)	0.7 (0.6-0.8)
II	8.2 (7.9-8.5)	3.3 (3.0-3.5)	1.2 (1.1-1.4)	0.7 (0.6-0.8)	1.8 (1.7-2.0)
III	3.7 (3.5-3.9)	1.5 (1.3-1.6)	0.7 (0.6-0.8)	0.4 (0.3-0.5)	0.7 (0.6-0.8)
IV	1.1 (1.0-1.2)	0.4 (0.4-0.5)	0.2 (0.1-0.2)	0.1 (0.1-0.2)	0.2 (0.2-0.3)
Grade					
Low	7.5 (7.2-7.8)	4.7 (4.4-4.9)	1.1 (1.0-1.3)	0.3 (0.3-0.4)	0.3 (0.3-0.4)
High	10.2 (9.8-10.6)	2.9 (2.7-3.1)	1.6 (1.4-1.7)	1.2 (1.1-1.3)	3.2 (3.0-3.4)
Tumor size					
≤2 cm	7.3 (7.0-7.7)	3.6 (3.4-3.8)	1.1 (1.0-1.2)	0.5 (0.4-0.6)	1.1 (1-1.2)
> 2 cm	10.4 (10.0-10.8)	4.0 (3.8-4.2)	1.6 (1.5-1.8)	0.9 (0.8-1.1)	2.4 (2.2-2.5)
Microinvasion	0.2 (0.1-0.2)	^c^	^c^	^c^	^c^
Diffuse	0.1 (0.1-0.2)	^c^	^c^	^c^	^c^
Lymph node involvement					
No	9.3 (9.0 - 9.7)	3.9 (3.7-4.2)	1.2 (1.1-1.3)	0.6 (0.5-0.7)	1.9 (1.8-2.1)
Positive	9.1 (8.8 - 9.5)	3.8 (3.6-4.0)	1.6 (1.4-1.7)	1.0 (0.9-1.1)	1.7 (1.5-1.8)
Prior cancer					
First primary	17.7 (17.2 - 18.2)	7.4 (7.1-7.7)	2.7 (2.5-2.9)	1.5 (1.4-1.7)	3.3 (3.1-3.6)
Non-first primary	1.9 (1.7 - 2.1)	0.7 (0.6-0.8)	0.3 (0.2-0.3)	0.2 (0.1-0.2)	0.4 (0.3-0.5)
Total	18.9 (18.4 - 19.4)	7.8 (7.5-8.2)	2.8 (2.6-3.0)	1.6 (1.5-1.8)	3.6 (3.4-3.8)

^a^Human epidermal growth factor receptor 2 (HER2), hormone receptor (HR), triple-negative (estrogen-receptor negative, progesterone-receptor negative, HER2^-^). ^b^95% confidence interval. ^c^Statistic not displayed because of fewer than 15 cases.

### Comparison of subtypes between age groups

**Figure 2:**
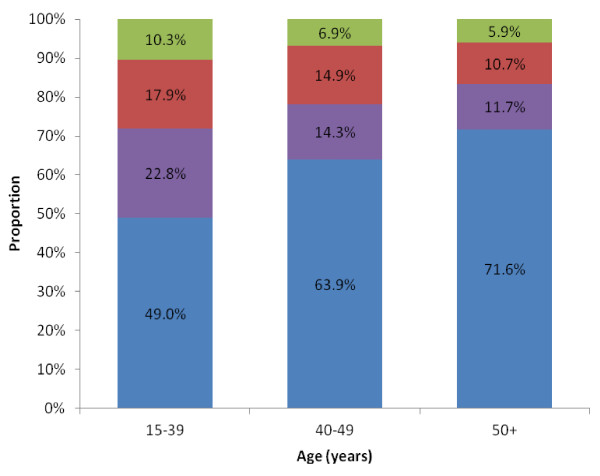
**Proportion of breast cancer subtypes among California women by age group, 2005-2009**. Hormone receptor (HR)-positive and human epidermal growth factor receptor 2 (HER2) negative (blue), HR^+^/HER2^+ ^(red), HR^-^/HER2^+ ^(green), and triple-negative (purple).

HR^+^/HER2^- ^was the most common subtype among all age groups, and HR^-^/HER2^+ ^was the least; however, the relative contribution of each subtype varied within age categories (Figure 2). HR^+^/HER2^- ^comprised an increasing proportion of all breast cancer diagnoses across the life span. By contrast, the relative contribution of HR^+^/HER2^+^, HR^-^/HER2^+^, and triple-negative decreased in successive age groups.

### Comparison of patient and clinical characteristics between age groups

Table 3 presents findings from logistic regression models comparing AYAs with women 40 to 49 years old and women older than 50 years separately to understand how characteristics differed among age groups. Compared with women older than 50 years and relative to Whites, Hispanics, Blacks, and Asians, AYAs were significantly more likely to be diagnosed with breast cancer and all breast cancer subtypes. Compared with women 40 to 49 years old, Hispanics (versus Whites) were more likely to be diagnosed with all subtypes, and Asians (versus Whites) were more likely to be diagnosed with HR^+^/HER2^- ^and HR^+^/HER2^+ ^subtypes. Black AYAs (versus Whites) were not significantly more likely than women 40 to 49 years old to be diagnosed with any subtype. AYAs also were significantly more likely to be diagnosed with stage III/IV disease and high-grade disease, except for HR^-^/HER2^+ ^breast cancer, than were older women. Compared with older women, AYAs were less likely to have had a prior cancer.

**Table 3:** Odds ratios^a ^for breast cancer diagnosis (15 to 39 versus 40 to 49 and 50+ years of age), by subtype^b^

	Total		HR^+^/HER2^-^		HR^+^/HER2^+^		HR^-^/HER2^+^		Triple-negative	
Characteristics	15-39/40-49	15-39/50+	15-39/40-49	15-39/50+	15-39/40-49	15-39/50+	15-39/40-49	15-39/50+	15-39/40-49	15-39/50+
	**OR (95% CI)**^c^	OR (95% CI)	OR (95% CI)	OR (95% CI)	OR (95% CI)	OR (95% CI)	OR (95% CI)	OR (95% CI)	OR (95% CI)	OR (95% CI)
Race/ethnicity										
White	Reference	Reference	Reference	Reference	Reference	Reference	Reference	Reference	Reference	Reference
Hispanic	1.58 (1.47-1.69)	3.25 (3.04-3.45)	1.59 (1.43-1.77)	3.23 (2.92-3.58)	1.51 (1.25-1.82)	2.92 (2.46-3.48)	1.36 (1.06-1.75)	2.63 (2.1-3.29)	1.52 (1.29-1.8)	3.26 (2.8-3.79)
Asian	1.32 (1.21-1.44)	2.47 (2.28-2.67)	1.52 (1.34-1.73)	3.00 (2.67-3.38)	1.30 (1.04-1.62)	2.26 (1.84-2.77)	1.00 (0.73-1.36)	1.44 (1.09-1.9)	1.26 (0.99-1.62)	1.96 (1.57-2.45)
Black	1.13 (1.00-1.27)	1.69 (1.52-1.89)	1.15 (0.94-1.40)	1.66 (1.38-2.00)	1.17 (0.86-1.58)	1.82 (1.37-2.42)	1.37 (0.90-2.09)	1.70 (1.18-2.46)	0.94 (0.74-1.19)	1.38 (1.11-1.71)
AJCC stage at diagnosis										
I/II	Reference	Reference	Reference	Reference	Reference	Reference	Reference	Reference	Reference	Reference
III/IV	1.33 (1.24-1.43)	1.58 (1.47-1.68)	1.38 (1.24-1.55)	1.82 (1.64-2.02)	1.51 (1.26-1.80)	1.65 (1.40-1.94)	1.22 (0.97-1.54)	1.44 (1.17-1.76)	1.35 (1.13-1.60)	1.40 (1.20-1.63)
Grade										
Low	Reference	Reference	Reference	Reference	Reference	Reference	Reference	Reference	Reference	Reference
High	1.82 (1.71-1.94)	2.56 (2.41-2.71)	1.75 (1.59-1.93)	2.37 (2.16-2.60)	1.29 (1.10-1.52)	1.59 (1.37-1.85)	0.85 (0.65-1.11)	1.05 (0.83-1.32)	1.53 (1.21-1.95)	2.87 (2.31-3.55)
Prior cancer										
1^st ^Primary	Reference	Reference	Reference	Reference	Reference	Reference	Reference	Reference	Reference	Reference
Non-1^st^ primary	0.72 (0.64-0.81)	0.32 (0.29-0.36)	0.71 (0.59-0.85)	0.29 (0.24-0.35)	0.84 (0.60-1.17)	0.33 (0.24-0.44)	0.57 (0.37-0.90)	0.32 (0.21-0.48)	0.60 (0.46-0.78)	0.41 (0.32-0.51)

^a^Adjusted for year at diagnosis, and the other variables in the table. ^b^Human epidermal growth factor receptor 2 (HER2), hormone receptor (HR), triple-negative (estrogen-receptor negative, progesterone-receptor negative, HER2^-^). ^c^Odds ratios (95% confidence intervals).

## Discussion

Our population-based study is the first to detail subtype-specific breast cancer occurrence among AYAs, aged 15 to 39 years. Similar to older women, AYAs were most likely to be diagnosed with the HR^+^/HER2^- ^subtype of breast cancer, followed by the triple-negative, HR^+^/HER2^+ ^and HR^-^/HER2^+ ^subtypes. However, compared with older women, AYAs had higher proportions of HR^+^/HER2^+^, triple-negative, and HR^-^/HER2^+ ^breast cancer subtypes and higher proportions of patients of non-White race/ethnicity. We also found that subtype distributions differed by race/ethnicity in AYAs, with Blacks and Hispanics less likely to be diagnosed with the HR^+^/HER2^- ^subtype, and Asians less likely and Blacks more likely to be diagnosed with the triple-negative subtype, as compared with Whites. We also found that AYAs were more likely to be diagnosed with stage III/IV disease and high-grade tumors than were older women.

Although previous studies in the CCR have found higher proportions of triple-negative [4,6,8] and HR^-^/HER2^+ ^[4,6] subtypes in younger women, ours is the first study to report a higher incidence of the HR^+^/HER2^+ ^subtype in AYAs compared with older age groups. This distinction is important because HR^+^/HER2^+^, like the triple-negative and HR^-^/HER2^+ ^subtypes, may be associated with worse survival than the HR^+^/HER2^- ^subtype [10,18]. The lower proportion of HR^+^/HER2^- ^breast cancer in AYAs compared with older women in our study may contribute to the poor prognosis reported for AYA breast cancer patients as a whole [3].

Poor prognoses among AYAs are particularly relevant to Black, Hispanic, and Asian women, who comprised a larger proportion of young compared with older breast cancer patients. Our results are consistent with previous reports of a higher incidence of breast cancer, especially triple-negative breast cancer [7,8], in young Blacks and relatively lower rates with increasing age for Hispanic and Asian women compared with Whites [19,20]. Black women have a high burden of triple-negative breast cancer, which is associated with poorer outcomes after breast-conserving therapy compared with other subtypes [21]. In contrast, Asian 15 to 39 year-olds had a much lower incidence of triple-negative breast cancer, consistent with our previous analyses [4,6]. We did not find an excess of breast cancer among Asians, in contrast to another California study using different methods of rate calculation [22]. Black and Hispanic women younger than 35 years have been found to have a poorer survival than White women [23], a finding that could be due, in part, to the lower proportion of HR^+^/HER2^- ^cancer in Black and Hispanic AYAs and a higher proportion of triple-negative breast cancer in Blacks.

Risk factors for breast cancer when treated as a single entity have been reported to differ by age [24], race/ethnicity [25,26], and hormone-receptor status [27-31]. Multiparity [24], prior mantle radiation for Hodgkin lymphoma [32], oral contraceptive use [33], and a low body mass index [34] are associated with breast cancer in young women and may vary by race/ethnicity [35,36]. Although studies have found that White women are more likely to experience risk factors for postmenopausal breast cancer, similar associations based on race/ethnicity are less clear for young women and should be the focus of future research. Given the differences observed in subtype distribution by age, it is probable that subtype-specific risk factors explain the heterogeneity of risk observed for breast cancer as a single entity.

Risk factor heterogeneity also has been reported by tumor ER and PR status [29,37] and, more recently, for HER2 status [11,29]. In pooled analyses, reproductive risk factors (age at menarche, parity, and age at first birth) and body mass index were associated with risk of HR positive, but not triple-negative tumors [31]. The only risk factor associated with the development of triple-negative tumors was family history, which was associated positively with all subtypes [31]. The Women's Health Initiative also reported appreciable differences among postmenopausal women for ER-positive versus triple-negative breast cancers with regard to risk factor associations with reproductive history and oral contraceptive use [28], but not body size or physical activity [27]. Future studies should continue to include information on HER2 status and also address how risk factors are associated with breast cancer subtypes, while considering age and race/ethnicity.

Genetic or other non-environmental contributions may explain the higher incidence of triple-negative breast cancer among Black women than White women [30]. A greater proportion of early breast cancer diagnoses are associated with germline mutations of BRCA1/2 [38] and TP53 [39]. BRCA1 mutations are associated with triple-negative breast cancer [40], which may contribute to the early age distribution of this subtype. Efforts to determine the prevalence of BRCA1/2 mutations in White versus Black populations, however, have been mixed [30,41]. Li Fraumeni syndrome, due to TP53 mutations, also is more prevalent in early-onset HR^-^/HER2^+ ^breast cancer [42], but the incidence of this disease is not known to differ by race/ethnicity [39].

Compared with older women, AYAs in our study were more likely to be diagnosed with stage III/IV and higher grade breast cancer and less likely to have been diagnosed with a previous cancer. Our multivariable-adjusted stage and grade results are consistent with results from univariate analyses in smaller studies [3,43]. Given the lack of screening mammography advisements for AYAs, whose diagnoses tend to follow identification of a palpable mass [44], it is not surprising that these women would present with later stage, higher grade disease. Despite the expectation that AYAs are more likely to have genetic syndromes that predispose them to cancer [39,41], we found that older women were more likely to have a prior cancer; presumably because older women had more time to acquire cancer-causing mutations.

Our study is the first, to our knowledge, to use population-based registry data for which ER, PR, and HER2 status are sufficiently complete to present breast cancer incidence rates in AYAs for the four major molecular subtypes by race/ethnicity. Another strength of our study is the relatively low percentage (16%) of women whose breast cancers were unclassified because of missing ER, PR, or HER2 receptor information compared with prior analyses [7,8]. To maximize the availability of HER2-receptor status, however, our study was restricted to diagnoses between 2005 and 2009, limiting the number of AYAs included. Although the reliability of ER and PR tests can be controversial [45], evidence suggests that results from a centralized pathology laboratory agree with registry reports for ER and PR status [46]. However, HER2 testing between community-based hospitals and centralized reference laboratories has been found to contain some disagreement [47]. Consensus-based methods to improve laboratory assays will continue to increase the reliability of ER, PR, and HER2 tests [48,49]. As with other studies that consider breast cancer subtype according to receptor status, we may be limited in that subtypes determined by ER, PR, and HER2 receptor status serve only as a proxy for full genetic profiling. These ER, PR, and HER2 designations, however, guide clinical treatment [50] and are becoming increasingly useful in epidemiologic research [4,10,21,29]. Our study is also subject to the potential misclassification of race/ethnicity, although we have detected excellent overall agreement with self-reported race/ethnicity for Whites and Blacks, and intermediate agreement for Hispanics and Asians [51,52].

## Conclusions

Our study adds to the evidence that AYA women with breast cancer have larger proportions of HR^+^/HER2^+^, HR^-^/HER2^+^, and triple-negative subtypes as compared with older women. Compared with White AYAs, Black and Hispanic women had lower incidence rates of HR^+^/HER2^- ^cancer, whereas Black women had higher rates and Asians had lower rates of triple-negative breast cancer. In addition, AYAs are more likely to be of Black, Hispanic, or Asian than of White race/ethnicity and diagnosed with stage III/IV and high-grade disease compared with older women. The subtype distributions may explain the poorer breast cancer survival previously observed in AYAs. Additional research is needed to understand more fully the racial/ethnic differences in breast cancer subtypes among AYAs.

## Abbreviations

AYA: adolescent and young adult; CCR: California Cancer Registry; CI: confidence interval; ER: estrogen receptor; HER2: human epidermal growth factor receptor 2; HR: hormone receptor; IHC: immunohistochemistry; IRR: incidence rate ratio; OR: odds ratio; PR: progesterone receptor.

## Competing interests

The authors declare that they have no competing interests.

## Authors' contributions

DJP performed the statistical analysis. DJP, AWK, and CAC participated in the interpretation of data, and in the drafting and critical review of the manuscript. THMK and MCD designed the study, interpreted the data, and led the writing and review of the manuscript. All authors read and approved the final manuscript.
